# Parasites that change predator or prey behaviour can have keystone effects on community composition

**DOI:** 10.1098/rsbl.2013.0879

**Published:** 2014-01

**Authors:** Melanie J. Hatcher, Jaimie T. A. Dick, Alison M. Dunn

**Affiliations:** 1 School of Biology, University of Leeds, Leeds, West Yorkshire, UK; 2 School of Biology, University of Bristol, Bristol, UK; 3 IGPS, School of Biological Sciences, Queen's University Belfast, Belfast, UK

**Keywords:** indirect interaction, intraguild predation, invasion, parasite-mediated, trait-mediated

## Abstract

Parasites play pivotal roles in structuring communities, often via indirect interactions with non-host species. These effects can be density-mediated (through mortality) or trait-mediated (behavioural, physiological and developmental), and may be crucial to population interactions, including biological invasions. For instance, parasitism can alter intraguild predation (IGP) between native and invasive crustaceans, reversing invasion outcomes. Here, we use mathematical models to examine how parasite-induced trait changes influence the population dynamics of hosts that interact via IGP. We show that trait-mediated indirect interactions impart keystone effects, promoting or inhibiting host coexistence. Parasites can thus have strong ecological impacts, even if they have negligible virulence, underscoring the need to consider trait-mediated effects when predicting effects of parasites on community structure in general and biological invasions in particular.

## 1. Introduction

There is an increasing realization that parasitism can play as pivotal a role as predation in structuring biological communities, often via indirect interactions with non-host species [1,2]. Indirect interactions occur when the impact of one species on another affects populations of a third species; classically, changes in population densities have been regarded as the main mechanism underlying these interactions. However, indirect interactions can also be driven by trait changes, which may be as important for community structure and function [3–5].

Trait-mediated interactions may be particularly relevant in parasite–host systems because parasites frequently modify host behaviour or physiology [6,7] and have been implicated as drivers behind a range of biological invasions, including wild oat (*Avena fatua*) in California, fire ants (*Solenopsis invicta*) in North America and amphipod crustaceans (*Gammarus*) in UK freshwaters [6,8]. Native/invader interactions for many species are governed by mutual intraguild predation (IGP), whereby potential competitors consume each other [9]. The invasive amphipod *Gammarus pulex* is a strong intraguild predator; however, parasitic infection alters both attack rates for intraguild predators and consumption of intraguild prey. For instance, *Echinorhynchus truttae* (Acanthocephala) infection increases maximal predation rates (functional responses) of *G. pulex* on native prey by 30% [10], but IGP on the native *G. duebeni* is nearly halved (prey mortality data [8]). *Pleistophora mulleri* (Microspora) infection of native *G. duebeni* reduces predation on smaller invasive *G. tigrinus* two- to threefold but doubles their vulnerability to predation by *G. pulex* [11].

Similarly, trematode-infected snails (*Littorina littorea*) exhibit 37.5% reduction in grazing pressure, influencing algal community composition [12], and barley yellow dwarf virus-infected bunchgrasses (*Nasella pulchra*) have more than 50% lower biomass, influencing competition with invasive species despite little infection-induced mortality [13]. The community consequences of parasite-induced trait-mediated effects have not, to our knowledge, been explored theoretically [6]. Predator–prey and host–parasitoid models demonstrate that trait-mediated indirect interactions can have strong and often counterintuitive impacts on populations and community structure [3,5]. Classical population models rarely consider trait-mediated effects; one way these can be incorporated is by modifying coefficients associated with trait parameters. The indirect effects of such trait changes on other species then emerge on examination of their population dynamics; we use this approach to examine how parasites altering two predation traits, *appetite* (predation rate) and *vulnerability* (to predation), influence population dynamics and community composition for two species engaged in IGP.

## 2. Material and methods

We develop a continuous time two host/one microparasite model based on the *Gammarus pulex/Gammarus d. celticus* system but broadly applicable to other invertebrate host–microparasite systems [10,11]. Parameters for competition and predation are provided by *G. pulex/G. d. celticus*, with others varied to allow sensitivity analysis and maintain generality (table 1). We model a microparasite with density-dependent parasite transmission [16] and for generality we examine three cases: parasites infect one of the intraguild predator–prey pair only (the case for *P. mulleri* in *G. d. celticus*); both species host the parasite, but only one experiences trait changes; or infection and trait changes occur in both species. As for *G. pulex/G. d. celticus*, we assume mutual asymmetric IGP, the species with higher *per capita* predation rate denoted IGpredator, and the weaker predator termed IGprey. IGpredator and IGprey also engage in cannibalism, often associated with IGP and frequent in *Gammarus* ([15]; table 1). The parasite can impart density (mortality) effects on infected hosts (as in [14]). We move on to include two parasite-induced trait effects, such that instantaneous rates of attack by intraguild predators (appetite) and consumption of intraguild prey (vulnerability) depend on infection, using symbolic constants to scale predation by or on the infected class.

**Table 1.** Terms in equations. Parasite-induced trait effects on IGP were included by scaling instantaneous predation rates on or by the infected subclass by *ρ_i_* (appetite) and *υ_i_* (vulnerability), assuming predation in infected–infected encounters is determined by predator appetite (appetite has priority over vulnerability). Parameter subscripts: 1, IGprey; 2, IGpredator.

parameter/variable (*units*)	definition	values taken (reference)
*S*_i_, *I*_i_ state variables (*per area*)	densities of susceptible and infected subpopulations, respectively, of host species *i*	n.a.
*N_i_* state variable (*per area*)	total population density, species *i*; initial population *N_i_* = 10 (*S_i_* = 9, *I_i_* = 1 or *S_i_* = 10, *I_i_* = 0) iterated to equilibrium	n.a.
*r_i_* (*t^−^*^1^)	intrinsic *per capita* population growth rate	*r*_1_ varied, *r*_2_ = 1.0 (reference values: [14])
*α_ij_* (*unitless*)	competition coefficient (the effect on species *i* of species *j*)	*α*_11_ = *α*_22_ = 0.005, *α*_12_ = *α*_21_ = 0.0005 [15]
*e* (*unitless*)	conversion efficiency of victims of predation or cannibalism into offspring	0.3 [15]
*γ_ij_* (*per predator–prey encounter · t^−^*^1^)	instantaneous rate of predation on species *i* by species *j* (before trait modification); subscripts 1 = IGprey, 2 = IGpredator	*γ*_12_ = 0.015, *γ*_21_ = 0.01 reflecting mutual asymmetric IGP [15]
*k* (*per encounter · t^−^*^1^)	instantaneous rate of cannibalism	0.01 [15]
*Ω_i_* (*per infection · t^−^*^1^)	*per capita* rate of parasite-induced mortality	0 ≤ *Ω_i_,Ω_j_* ≤ 0.5 (as given, figure 1)
*β_ij_* (*per infectious–susceptible encounter*)	parasite transmission efficiency to species *i* from species *j*	*β*_11_ = *β*_22_ = 0.05, *β*_12_ = *β*_21_ = 0.005
*υ*_*i*_ (*unitless*)	vulnerability trait modifier (scales predation on infected subclass of species *i* by susceptibles of *j*)	0 ≤ *υ_i_* ≤ 2 (applied to *γ*_1*I*2*S*_, *γ*_2*I*1*S*_)
*ρ_i_* (*unitless*)	appetite trait modifier (scales predation by infected subclass of species *i* on infected and susceptible subclasses of *j*)	0 ≤ *ρ_i_* ≤ 2 (applied to *γ*_1*S*2*I*_, *γ*_1*I*2*I*_, *γ*_2*S*1*I*_, *γ*_2*I*1*I*_)

Terms in equation (2.1) (below) for uninfected (susceptible, *S*) hosts reflect three components of IGP: (i) interspecific competition (Lotka–Volterra form, normalized to obviate explicit carrying capacity, [14]), (ii) predation (linear function of IGpredator and prey densities weighted by coefficients of attack) and (iii) cannibalism (proportional to population density, weighted by coefficient of attack). To model trait-mediated effects, we further break down predation (square brackets) into interactions between infected/susceptible host classes with attack rates scaled to reflect changes in appetite (*ρ_i_*) and vulnerability (*υ_i_*) owing to infection. The infected class (*I*) suffers loss through parasite-induced mortality, cannibalism and IGP (equation (2.2)). Parasite transmission also causes loss of susceptibles (equation (2.1); terms with *β*) and gain of infecteds (equation (2.2)). We assume pure horizontal parasite transmission; hence reproduction by infected individuals yields susceptible offspring, so energetic gains from predation/cannibalism by infecteds accrue to the susceptible class (final term within square brackets, equation (2.1)). Changes in the population densities of the two host species (*i,j*) are thus2.1
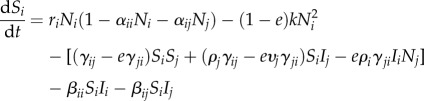
2.2
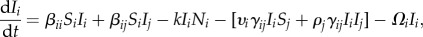
with structurally symmetric equations for species *j*. Equilibria were examined using numerical exploration of parametrized equations, with state transitions identified using binary search algorithms programmed in perl (see the electronic supplementary material).

## 3. Results

**Figure 1.**
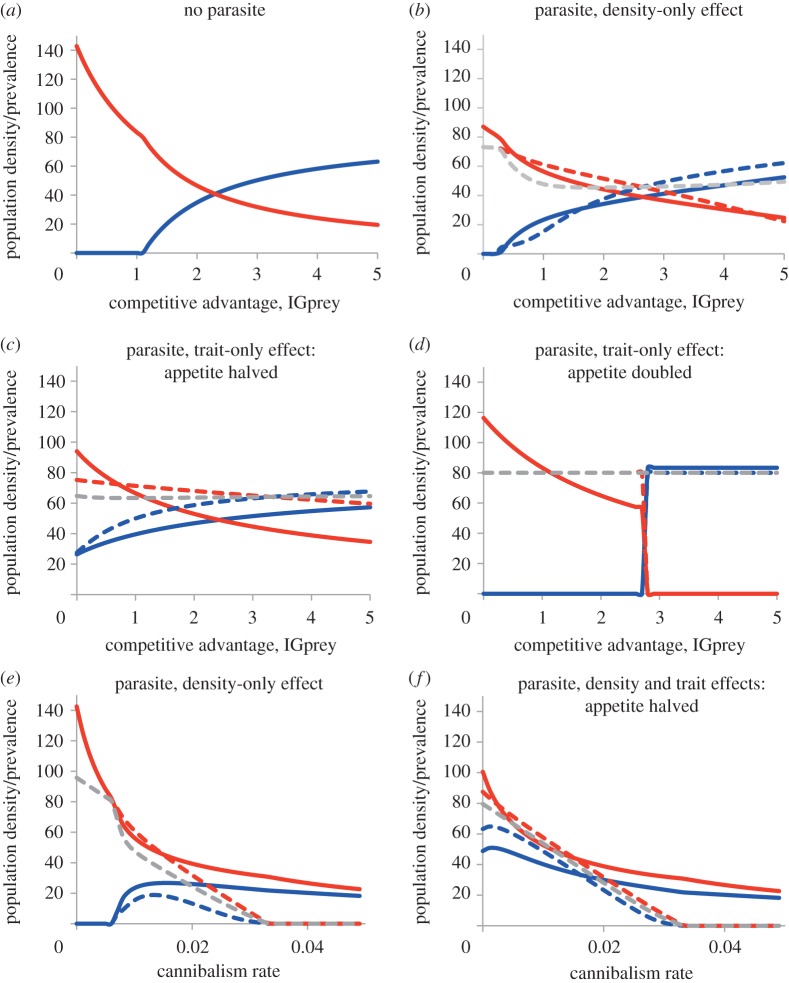
Effect of parasitism on community composition for IGP systems, with respect to relative competitive advantage of IGprey (intraspecific competition ratio, IGpredator : IGprey: *α*_2_*α*_2_/*α*_1_*α*_1_; *a–d*) and cannibalism (*k*_1_ = *k*_2_; *e,f*); (*a*) without parasite; (*b*) parasite with density-only (mortality) effects (*Ω*_1_ = 0.1, *Ω*_2_ = 0.3); (*c,d*) with trait-only effects (*c*: *ρ*_1_ = *ρ*_2_ = 0*.*5; *d*: *ρ*_1_ = *ρ*_2_ = 2*.*0; *Ω*_1_ = *Ω*_2_ = 0); (*e*) density-only effects (*Ω*_1_ = 0.1, *Ω*_2_ = 0.3); (*f*) density and trait effects (*Ω*_1_ = 0.1, *Ω*_2_ = 0.3, *ρ*_1_ = *ρ*_2_ = 0*.*5). Solid lines: equilibrium population density (m^−2^) (blue, IGprey; red, IGpredator); dashed lines: %parasite prevalence (blue, %prevalence in IGprey; red, %prevalence in IGpredator; grey, %prevalence across both hosts). Parameter subscripts: 1, IGprey; 2, IGpredator.

Three non-trivial equilibrium outcomes are possible in classical models of IGP: the IGpredator is excluded and the IGprey persists; the IGprey is excluded and the IGpredator persists; or both coexist. In the absence of parasitism, coexistence requires the superior intraguild predator to be the inferior competitor (figure 1*a*: IGprey are maintained once intraspecific competition between IGpredators exceeds that between IGprey; [9]). Parasitism can enhance IGpredator/prey coexistence, via density ([14], figure 1*b,e*) or trait (figure 1*c–f*) effects. Interestingly, trait effects (zero virulence) can have as pronounced an impact as density in promoting (figure 1*c,f*) or inhibiting (figure 1*d*) coexistence. Parasite prevalence depends on host competition and predation, and parasite-induced mortality (figure 1). Parasitism also interacts with cannibalism in determining population outcomes; cannibalism enhances IGpredator/IGprey coexistence [17], but reduces equilibrium population densities (figure 1*e,f*). Consequently, strong cannibalism eliminates the parasite by driving host populations below predicted thresholds for parasite establishment [16].

**Figure 2.**
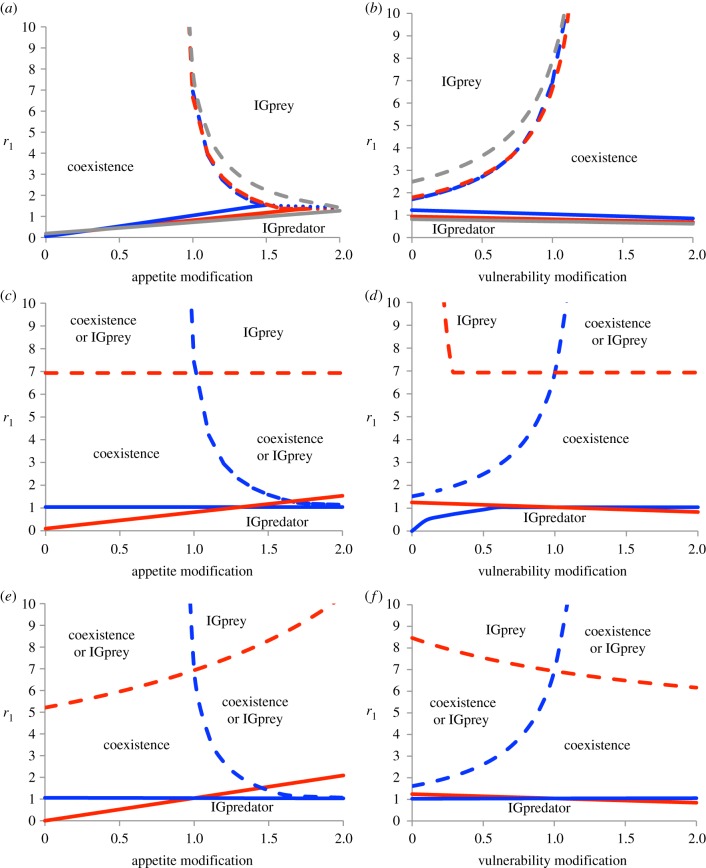
Impact of trait- and density-mediated indirect effects of parasitism on IGP, on boundaries between stable states in terms of *r*_1_ (reproductive rate of IGprey) given parasite effect on one trait (horizontal axis), with the second trait fixed. Lines show state boundaries for hosts/virulence (as coloured): dashed lines, coexistence-IGprey boundaries; solid, coexistence-IGpredator; dotted, IGprey–IGpredator. (*a,b*) Parasite infects both species and modifies traits symmetrically (red, *Ω*_1_ = 0.1, *Ω*_2_ = 0.3; blue, *Ω*_1_ = *Ω*_2_ = 0; grey, *Ω*_1_ = *Ω*_2_ = 0.5); (*c,d*) parasite infects one species (blue, IGprey as host; red, IGpredator host); (*e,f*) parasite infects both species but modifies traits of only one (blue, IGprey affected; red, IGpredator affected; virulence in *c–f*, *Ω*_1_ = *Ω*_2_ = 0). Parameter subscripts: 1, IGprey; 2, IGpredator.

Phase boundaries for coexistence are contingent on trait modification, and also the host species infected or affected (figure 2). Parasites that reduce predatory appetite (figure 2*a*) or increase vulnerability to predation (figure 2*b*) enhance coexistence. Again, avirulent parasites inducing *only* trait effects have similar impact to virulent parasites (figure 2*a,b*). Hence, parasites that alter host traits can have clear keystone effects even if they are relatively benign, enhancing the range of conditions for IGP persistence, or excluding the IGpredator or IGprey.

Qualitatively similar patterns occur for different host/transmission scenarios (figure 2; electronic supplementary material, figures S1 and S2), but parasites confined to one-host species yield somewhat different patterns. For instance, the point at which IGpredators are eliminated is independent of effects on their appetite (figure 2*c*), because IGpredators become too rare approaching this boundary to sustain parasite populations. Similarly, elimination of IGprey is independent of effects on IGprey appetite (figure 2*c*). By contrast, vulnerability influences transitions even for rare hosts (figure 2*d*): reduced vulnerability is advantageous to prey; parasites inducing such changes effectively enhance host fitness, reducing the threshold required for their own maintenance [16]. When parasites infect both species, all boundaries are trait-dependent because the parasite is maintained between both hosts (figure 2*e,f*).

## 4. Discussion

Parasites, and their trait-mediated effects, are implicated in driving numerous aquatic and terrestrial invasions [6,8], often in association with IGP [18,19]. Trait-mediated indirect interactions with parasites could explain why IGP, a common ecological interaction in natural communities, persists despite theory that paradoxically concludes its persistence unlikely [9,19]. Trait changes may be particularly relevant in invasive systems (and under ecological change generally), where invasive and native species meet novel biotic and environmental conditions conducive to trait shifts [6].

Our analysis shows that the trait-mediated effects of parasites not only alter host coexistence outcomes; they can have stronger impacts on host communities than density-mediated effects. Outcomes depend on community context and mechanism, including trait(s) altered, host(s) affected or infected, and host trophic position (figure 2). Conceivably, many traits (e.g. competitive or cannibalistic) might be altered by parasitism and other interactions may be influenced by such trait effects [5].

Considering how multiple trait effects combine is a pressing area for future research [5–7]. Golubski & Abrams [20] argue that trait modifiers usually interact antagonistically, in part due to constraints on trait plasticity. In our model, the traits examined do indeed influence predation rate in opposition, but their combined influence on community structure is mechanism- and context-dependent. Propagation of trait (or density) effects of parasitism depends on interactions with other species; the community consequences of such potentially bidirectional interactions are unclear [2]. For instance, cannibalism alone theoretically enhances IGP persistence [17], but parasites are lost from strongly cannibalistic populations (figure 1*e,f*); how these processes interact warrants further study.

Parasite-induced changes in appetite and vulnerability are documented for a variety of systems [8]; these effects are not well-addressed by classical concepts of virulence, traditionally defined in terms of host mortality. By definition [4,5], trait-mediated indirect effects emerge only in the context of population or community interactions and cannot easily be deduced from study of isolated, focal hosts. Such ‘cryptic virulence’ [8,11] is increasingly recognized in ecology [6–8]. Within parasitology, some cases are well studied (e.g. host manipulation in relation to transmission strategy; [7]) but the broader epidemiological and evolutionary ramifications of extended concepts of virulence have yet to be examined.

Our results highlight the need to consider trait-mediated indirect interactions in predictive management of invasions and biocontrol scenarios. The inclusion of trait effects can make practical prediction difficult, particularly if systems lie close to phase boundaries. Failure to consider trait-mediated indirect effects in risk assessment for biocontrol agents or potential invasive species could lead to erroneous predictions as to their efficacy or impact.
